# A Reversible Optical Sensor Film for Mercury Ions Discrimination Based on Isoxazolidine Derivative and Exhibiting pH Sensing

**DOI:** 10.3390/bios12111028

**Published:** 2022-11-16

**Authors:** Reham Ali, Siwar Ghannay, Sabri Messaoudi, Fahad M. Alminderej, Kaïss Aouadi, Sayed M. Saleh

**Affiliations:** 1 Department of Chemistry, College of Science, Qassim University, Buraidah 51452, Saudi Arabia; 2 Chemistry Department, Faculty of Science, Suez University, Suez 43518, Egypt; 3 Faculty of Sciences of Bizerte, Carthage University, Bizerte 7021, Tunisia; 4 Faculty of Science of Monastir, University of Monastir, Avenue of the Environment, Monastir 5019, Tunisia; 5 Chemistry Branch, Department of Science and Mathematics, Faculty of Petroleum and Mining Engineering, Suez University, Suez 43721, Egypt

**Keywords:** optical sensor, pH, Hg(II), ESIPT, ratiometric, DFT

## Abstract

We developed a new optical sensor for tracing Hg(II) ions. The detection affinity examines within a concentration range of 0–4.0 µM Hg(II). The sensor film is based on Methyl 2-hydroxy-3-(((2S,2’R,3a’S,5R)-2-isopropyl-5,5’-dimethyl-4’-oxotetrahydro-2’H-spiro[cy-clohexane-1,6’-im-idazo[1,5-b]isoxazol]-2’-yl)methyl)-5-methylbenzoate (IXZD). The novel synthesized compound could be utilized as an optical turn-on chemosensor for pH. The emission intensity is highly enhanced for the deprotonated form concerning the protonated form. IXZD probe has a characteristic fluorescence peak at 481 nm under excitation of 351 nm with large Stocks shift of approximately 130 nm. In addition, the binding process of IXZD:Hg(II) presents a 1:1 molar ratio which is proved by the large quench of the 481 nm emission peak of IXZD and the growth of a new emission peak at 399 nm (blue shift). The binding configurations with one Hg(II) cation and its electronic characteristics were investigated by applying the Density Functional Theory (DFT) and the time-dependent DFT (TDDFT) calculations. Density functional theory (DFT) and the time-dependent DFT (TDDFT) theoretical results were provided to examine Hg(II)-IXZD structures and their electronic properties in solution. The developed chemical sensor was offered based on the intramolecular charge transfer (ICT) mechanism. The sensor film has a significantly low limit of detection (LOD) for Hg(II) of 0.025 μM in pH 7.4, with a relative standard deviation RSD_r_ (1%, *n* = 3). Lastly, the IXZD shows effective binding affinity to mercury ions, and the binding constant K_b_ was estimated to be 5.80 × 10^5^ M^−1^. Hence, this developed optical sensor film has a significant efficiency for tracing mercury ions based on IXZD molecule-doped sensor film.

## 1. Introduction

Heavy metal contamination of water resources poses a significant danger to human health and the surrounding environment and causes many associated risks. Despite the efforts to overcome this problem, the industrial boom and multiple human activities led to a steady increase in pollution caused by heavy metals in various water resources. Thus, many global scientific organizations set limits for different concentrations of heavy metal ions. Therefore, many efforts are being introduced to detect the concentrations of various heavy metal ions, involving lead, mercury, arsenic, nickel, and cadmium, to mask the severe health influences of bio-accumulation and bio-magnification [1,2,3,4,5].

Mercury is one of the dangerous heavy metal ions, considered the most toxic element because of its great danger to health, including heart muscle diseases, kidney toxicity, neurotoxicity, tendency to produce cancer, autoimmune diseases, and male reproductive toxicity [6,7,8,9,10]. Additionally, the surrounding environment is affected by the toxicity of mercury, which results mainly from the handling of many different chemicals and from the other processes that take part in the marine and aquatic environments and the conversion of mercury into various organic and inorganic forms. By auditing, microorganisms can convert inorganic mercury into methylmercury, a conceivable neurolysin [11,12,13]. Thus, detecting mercury in the environment takes much of people’s attention. Memorably, many methods have been used to detect mercury ions such as inductive coupling plasma mass spectrometry, surface-enhanced Raman spectroscopy, and atomic absorption spectrometry, which are expensive and require extended time pretreatment. People with special training have to carry out these processes [14,15,16]. Chemical sensors display great efficiency in detecting mercury ions due to their biocompatibility, portability, and promise for use in field treatments [17,18,19,20,21,22].

Researchers developed and designed several significant optical sensors with extreme selectivity and sensitivity to determine several metal ions. Detection of heavy metal ions has been considered one of the essential remarkable and vital research streams in analytical chemistry. The benefits of the fluorescence technique for sensing involve crucial factors. First, molecular fluorescence is enormously sensitive; these measurements produce slight or no destruction to the host structure, thus delivering the capability for wholly non-intrusive sensing. Second, the information provided by the fluorescence techniques shows the system design and environmental behavior of molecules and the way in which these vary in reaction to analyte alterations in the surrounding environments. For example, some heavy metal-sensitive probes labeled to proteins can be quenched or enhanced as the adaptation variations reveal the dye to heavy metals. The biomolecule distribution in a particular environment and under such conditions can be investigated using fluorescence resonance energy transfer (FRET) [23,24]. In addition, the synthesis of isoxazolidines has become increasingly important because of their diverse biological activities [25,26,27]. Recently, we have described the synthesis of isoxazolidine derivatives which can be used as potential alternatives to modulating T2DM (type 2 diabetes mellitus) [28]. In addition, isoxazolidine is a precursor of many bioactive molecules such as amino acids [29], β-lactams [30], amino lactones [31], and alkaloids accessible by breaking the N-O bond of the isoxazolidine ring [32].

**Scheme 1:**
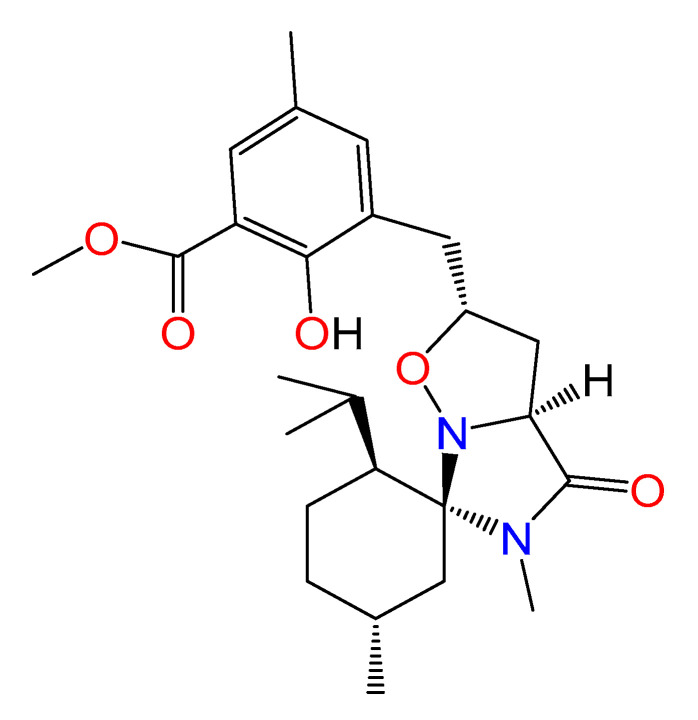
The chemical structure of IXZD molecule.

In this project, we developed a novel chemosensor utilizing IXZD for detecting Hg(II) ions, and this sensor is designed utilizing a substantial IXZD sensing molecule. The structure of the IXZD molecule is displayed in Scheme 1. Additionally, the novel probe can be used as a turn-on optical sensor for pH sensing as the luminescence is highly increased in the case of the deprotonated form concerning the protonated form. The proposed mechanism can be referred to as metal–ligand binding between IXZD and Hg(II) ions to introduce a metal complex. The explanation of the formed metal complex is based on an intramolecular charge transfer (ICT) [33,34,35]. The sensing process of mercury ions by IXZD probe was studied utilizing UV–Vis and emission intensities measurements. The new sensor provides considerable sensitivity and selectivity with a precious value of LOD and rapid reversibility for tracing mercury ions.

## 2. Materials and Methods

### 2.1. Chemicals

**Scheme 2:**
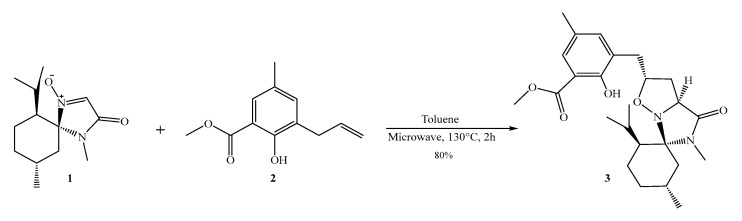
Preparation of the sensing probe IXZD.

All the fine chemicals were purchased from Sigma-Aldrich. All chemical solvents were analytical grade and used as received in all experiments. Methyl 2-hydroxy-5-methyl-3-(prop-2-en-1-yl)benzoate (Scheme 2) was purchased from Acros company https://www.acros.com/ (accessed on 11 November 2022). Stock solutions of the applied metal nitrates were prepared with bi-distilled water before experimentation and directly involved in the experimental series.

### 2.2. Instruments

The NMR spectrum of the sensing compound was measured utilizing a JEOL JNMECA 600 spectrometer (600-MHz for ^1^H and 150-MHz for ^13^C) regarding TMS, Tetramethylsilane as an internal standard. Melting points were detected based on a Kofler Microhot Stage Instrument. UV–Vis spectra were attained in a 1 cm quartz cell with an Evolution™_200-series/UV–Visible spectrophotometer. Fluorometric measurements, including excitation and emission spectra, were measured on a JASCO FP-6300 spectro-fluorometer in a 1 cm quartz cell.

### 2.3. Synthesis of IXZD

In a 10 mL glass vial, nitrone **1** (150 mg, mmol) was dissolved with methyl 2-hydroxy-5-methyl-3- (prop-2-en-1-yl) benzoate **2** in 6 mL of toluene [23]. The reaction has been activated by a mono-wave 200 system operating at 130 °C for 2 h. The obtained product has been purified by flash chromatography on silica gel (EtOAc/PE 2/8) to provide the cycloadduct 3, as shown in the following Scheme 2. The NMR spectrum is exhibited in Figure S1 (Supplementary Materials).

### 2.4. Optical Characteristics of IXZD

The UV–Vis spectra of 2 × 10^−3^ M IXZD were collected in 20 mM Britton–Robinson (BR) buffer, RB is a “universal” buffer for pH 2–12, and it consists of boric acid, phosphoric acid, and acetic acid that has been titrated to the desired pH with sodium hydroxide. Through titration, we fixed the total volume of the prepared solution of IXZD to 2.0 mL. The luminescence intensities of the prepared solutions were measured at different pH values utilizing the same conditions. The excitation and emission slit widths for all investigated solutions were 5 nm. A definite concentration of IXZD in an aqueous BR buffer (20 mM) was used for the luminescence titration to demonstrate the efficiency of the sensor at different pH. Various prepared solutions of BR buffer with a wide range of pH scales from 2 to 12.5 were used to investigate the pH sensing of the chemical sensor.

### 2.5. Design of the Optical Film

**Scheme 3:**
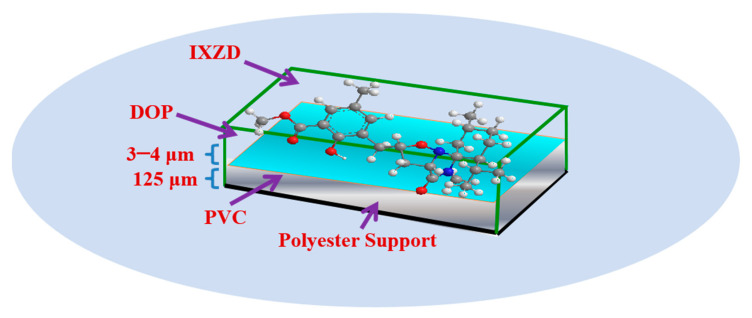
Schematic diagram of IXZD sensor film.

The sensing film was designed by introducing a cocktail solution. This solution consists of 2.0 mg IXZD, 25.4 mg of Polyvinyl chloride, and 51 mg of dioctyl phthalate plasticizer in 1.8 mL THF (Tetrahydrofuran). Moreover, the cocktail solution was mixed for 6 h. A knife coater spread the solution on a polyester polymer support [36]. The sensor film was left to dry in the air for 2 h. The estimated thickness of the sensor film was calculated from the cocktail quantity to be 3–4 μm after evaporation of the solvent, as exhibited in Scheme 3.

### 2.6. The Binding Study

The stoichiometric of the IXZD sensing probe and Hg(II) interaction was investigated by applying Job’s method [37,38]. The chemical binding was exploited in pH 7.4 buffer solution using a molar ratio of 1:1 of 1 × 10^−6^ M IXZD sensing compound and Hg(II) ions. The relation between the molar ratios of the reactants (9:1 to 1:9) against the luminescence intensities ratio of the IXZD probe at (399/481) was studied.

## 3. Results

### 3.1. The Optical Properties of IXZD

**Figure 1:**
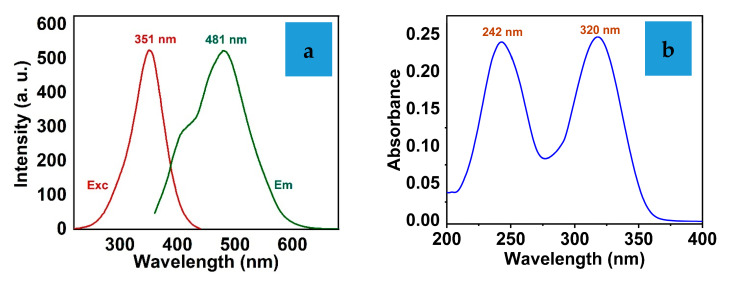
IXZD Optical Properties. (**a**) Fluorescence/excitation spectra under 351 nm excitation and (**b**) UV–Vis spectrum.

The optical measurements of the IXZD sensing probe were collected in DMSO: MeOH(5:95)-based BR buffer (20 mM) at physiological pH (7.4). The IXZD probe showed considerable properties, including a significant Stokes shift ≈ 130 nm as shown in Figure 1a. IXZD shows characteristic absorption peaks at 242 and 320 nm. Moreover, it displays a prominent fluorescence peak at 481 nm under excitation with 351 nm. ESIPT (Excited state intramolecular proton transfer) chromophores absorb photons in the UV region and emit energy in the visible region. The absorption and emission wavelengths are based predominantly on the molecular structure of the studied probes and their attached functional, active groups. Additionally, the absorption and emission maxima of the IXZD probe are independent of the concentration. This work uses the IXZD absorption spectra to demonstrate its efficiency as a pH chemical probe. The absorption spectra of IXZD were carried out at various pH values using 20 mM BR buffer solutions. Figure 1b exhibits the absorption spectra of IXZD; it shows two distinct peaks at 242 nm and 320 nm, which are assigned to the π–π * and *n*–π * transitions.

**Figure 2:**
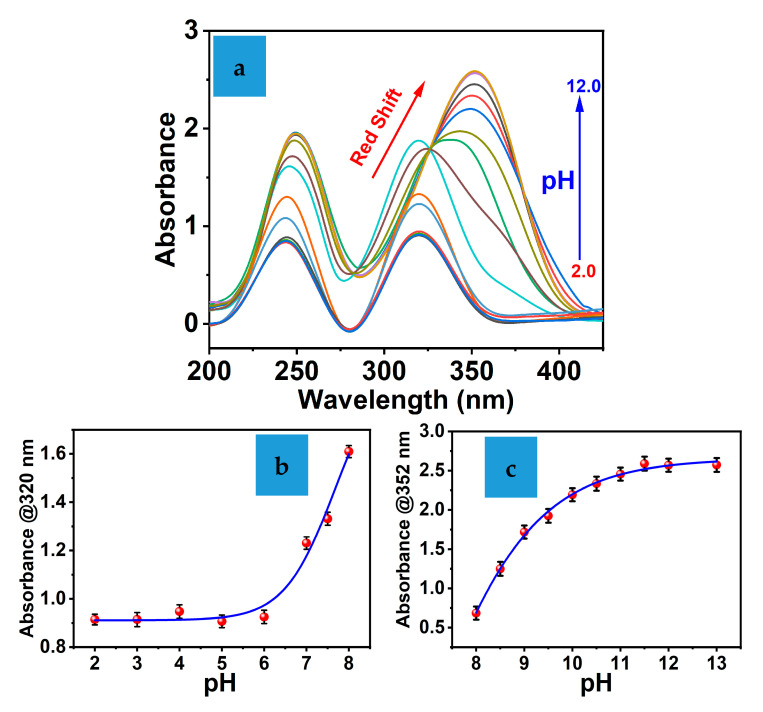
(**a**) Absorbance spectra of IXZD at various pH values; (**b**,**c**) the absorbance of the IXZD versus pH.

With the rising pH values of the studied solution containing 0.5 mg/mL IXZD, a continuing enhancement in the absorption maximum of the peak located at 320 nm is perceived, associated with the development of an additional intense absorption peak located at 352 nm with a distinctive isosbestic point at approximately 326 nm. The ESIPT mechanism is responsible for the red shift of the absorption band, and the absorbance increment of the new peak is due to the ICT mechanism of the phenoxide tautomer in the deprotonated IXZD anion structure that takes place in the ground state. Figure 2 exhibits the relation of the IXZD absorbance against the pH change of the investigated solution. Additionally, the inset of Figure 2 displays the calibration plot attained for the absorbance wavelengths at 320 nm and 352 nm of the IXZD probe versus the pH values of the studied solutions in the presence of 20 mM BR buffer. The harmonic relationship is established during the pH-titrimetric reaction, where the absorbance peak at 320 nm gradually shifts, and the second absorbance peak at 352 nm is synchronously enhanced.

**Figure 3:**
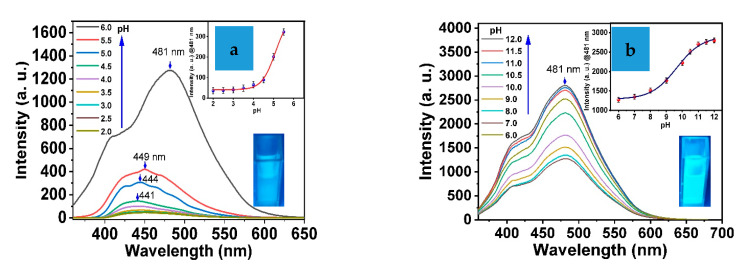
Emission spectra of IXZD at various pH values: (**a**) pH range (2–6); (**b**) pH range (6–12).

Figure 3 represents the influence of the pH on the luminescence intensities of IXZD. IXZD exhibits a weak emission peak centered at 441 nm in acidic pH. The emission maximum at 441 nm enhances and changes to 481 nm, red shift, by increasing the pH to six as shown in Figure 3a. This can be attributed to the ICT mechanism of the phenoxide tautomer in the deprotonated IXZD anion structure that occupies the ground state. By transferring the pH from the acidic medium to the basic medium, pH = 6–12, the enhancement of the emission intensities at 481 nm takes place (see Figure 3b). The inset graphs introduce the relation of IXZD fluorescence intensities at 481 nm against the pH of the studied solutions.

### 3.2. Study the pH Property of the Chemical Probe

In the present work, we introduce a novel type of pH sensor. The mechanism of the new chromophore involves the ESIPT mechanism. Additionally, the basic idea of the ESIPT mechanism depends on the excitation of the organic probe. A photoinduced proton tautomerization can introduce this from the enol form (E*) to the phototautomer keto form (K*) with the intramolecular hydrogen bond. This is induced by internal proton transfer from a hydroxyl group in the enol form to the oxygen atom that acts as a highly electronegative withdrawn atom. When the keto form relaxes into the ground state, the inverse proton transfer mechanism returns the desired enol structure. ESIPT luminescence mechanism delivers distinctive characteristics, including strong luminescence and a significant Stokes shift value, due to the fluorescence produced from K*. The mainly unique feature of IXZD is its luminescence performance. This chemical label is present in the enol tautomer structure in the ground state. During the excitation process, in the presence of a photon, a proton is moved from the hydroxyl donor functional group to the carbonyl acceptor group, which generates the excited state proton transfer tautomer. The constructed excited state then consumes its energy through the relaxation process to the lower energy ground state, escorted by luminescence. Remarkably, the energy level of the keto structure tautomer K* in the excited state, which presents based on the proton transfer, has less energy level than the enol structure tautomer E*. Additionally, the keto structure tautomer K in the ground state has a greater energy level than the enol structure tautomer E.

**Scheme 4:**
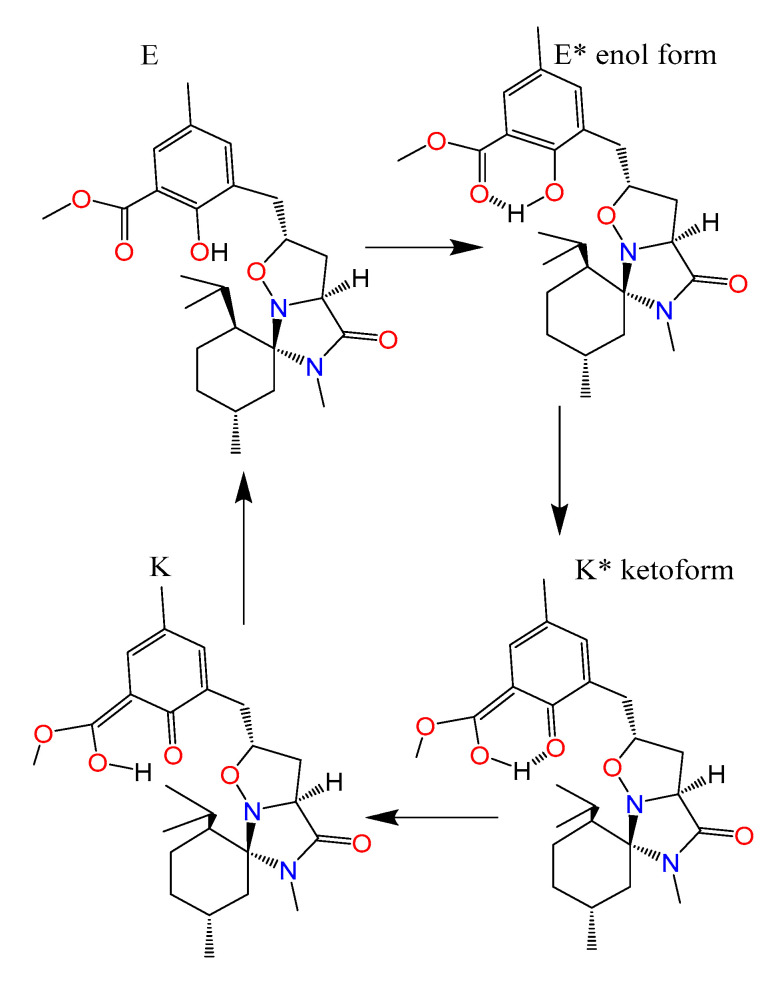

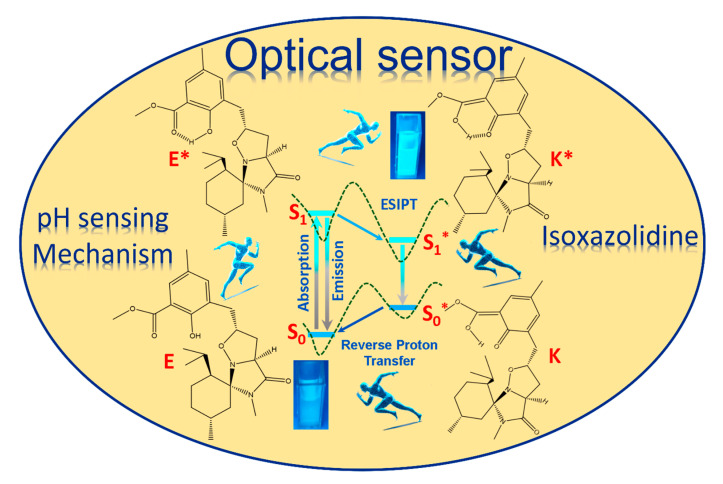
The ESIPT mechanism of IXZD.

As a result, the energy transfer from excited K* state to the ground k state is less than the energy released during the transformation from the excited E* state to the ground E state. Thus, the Stokes shift of the ESIPT IXZD chemical probe in keto form is more significant than that of the enol form (as shown in Scheme 4). Furthermore, Scheme 4 shows two sets of energy levels: S_0_ and S_0_*, which represent enol and keto tautomers that present at the ground states, and S_1_ and S_1_*, which represent the enol and keto tautomers that provide the first singlet excited states separately [34]. The asterisk denotes the tautomer that formed during proton transfer. The hypothesized enol tautomer processes show rapid enol tautomer structure relaxation from the excited to the ground state, accompanied by decreasing emission or a faster energy route involving hydrogen proton transfer from the enol form to the keto tautomer structures.

In the most common design of pH sensor, pH probes rely on weak acidic dyes whose dissociated and undissociated forms have different absorption or fluorescence in the pH range of interest. Although a great variety of pH indicators is known, only a few possess the requirements for use in a pH probe [39], including (a) a pK in the pH range of interest; (b) strong absorption within UV–Vis range, making them compatible with the excitation light sources; (c) photostability and chemical stability; (d) lack of toxicity especially when the probe embedded in a polymer film, and (e) the availability of functional groups suitable for chemical immobilization. In the case of fluorescence-based pH probes, large Stokes’ shifts and lack of quenchability by oxygen and other sample constituents are desirable.

### 3.3. Sensor Characteristics

**Figure 4:**
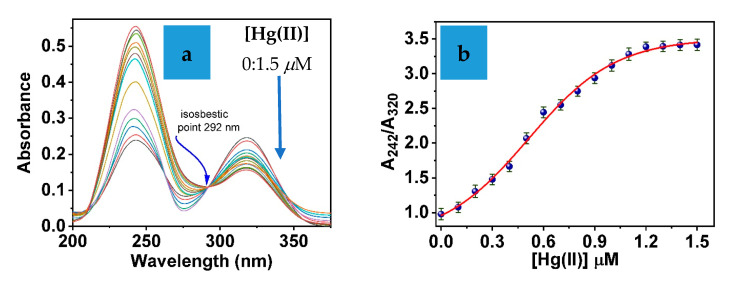
(**a**) Behavior of UV–Vis spectrum of IXZD in the presence of 0:1.5 μM Hg(II) and (**b**) A_242_/A_320_ absorbance ratio of IXZD against [Hg(II)].

The colorimetric and luminescence titration techniques were used to evaluate the IXZD molecule sensing capacity towards the mercury ions. The absorbance peak at 320 nm regularly decreased in the presence of Hg(II) ions. At the same time, the absorbance peak at 242 nm was enhanced. The ratiometric absorbances of the IXZD chemical probe in the presence of Hg(II) were noticed between these two significant peaks, with a considerable isosbestic point located at 292 nm, as shown in Figure 4a. Additionally, a significant curve-fit relation was introduced concerning the IXZD absorbance values ratio at 242 and 320 nm and the amount of Hg(II) ions between 0 and 1.5 µM (see Figure 4b).

Furthermore, the A_242_/A_320_ absorbances ratio of the IXZD is directly proportional to the concentration of Hg(II) ions up to a 1:1 molar ratio of ligand/metal. Compared to the UV–Vis spectrum of free IXZD, the quenching of the absorbance peak at 320 nm and the enhancement of the absorbance peak at 242 nm, respectively, prove the strong attendance of IXZD for the binding process-based IXZD-Hg(II) chelation. The disparities in the UV–Vis spectrum can be revealed by the significant IXZD chelated with Hg(II) because of the behavior of the conjugated electronic structure [40].

**Figure 5:**
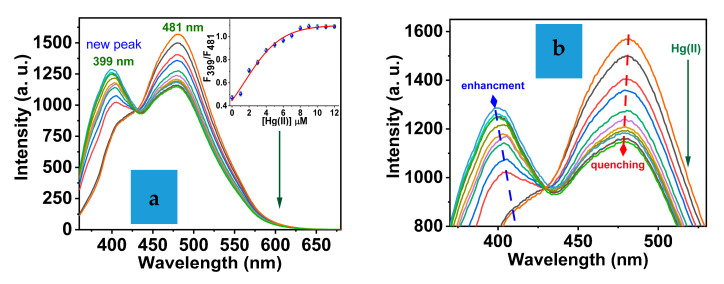
(**a**) Fluorescence titration of Hg(II)-IXZD with a molar ratio of 0:1 (metal: IXZD); inset figure represents the IXZD emission intensities ratio at 399 and 481 nm versus [Hg(II)]; (**b**) IXZD emission intensities scope at 399 nm and 481 nm, respectively.

The fluorescence titration of the IXZD sensing molecule versus Hg(II) concentrations was carried out in a 20 mM BR buffer solution at a pH of 7.4. The IXZD molecules exhibit a central emission peak at 481 nm under 351 nm excitation wavelength (Figure 5a). The binding of Hg(II) to the IXZD with a molar ratio of 0:1 sensing probe to Hg(II) ions immediately decreased the luminescence of the maximum peak IXZD at 481 nm with the growth of a new emission maximum 399 nm, indicating IXZD chelates mercury ions. The emission maximum at 481 nm was significantly decreased by introducing Hg(II) metal ions into the system, proposing additional proof of the chelation of Hg(II) ions by the IXZD sensing molecule. The IXZD emission did not change or exhibit any changes in the emission peaks on consistent addition of mercury ions ≈ 12 μM (inset Figure 5), confirming the binding process of Hg(II)-IXZD with an equivalent ratio of 1:1 (metal: IXZD sensing probe).

### 3.4. The Sensing Film

**Figure 6:**
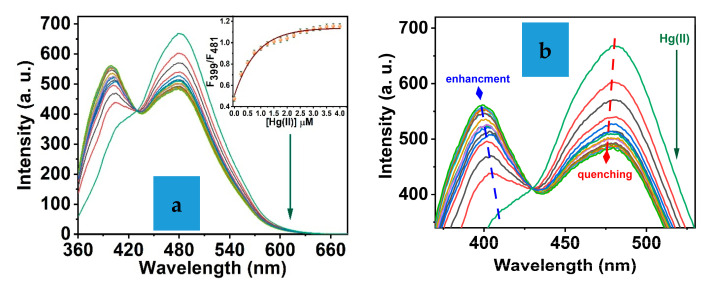
(**a**) Fluorescence titration of the sensor film; inset figure represents the IXZD emission intensities ratio at 399 and 481 nm versus [Hg(II)]; (**b**) IXZD emission intensities scope at 399 nm and 481 nm, respectively.

The response of sensor film towards Hg(II) was investigated. The fluorescence spectra of the sensor film in the presence of various [Hg(II)] were detected, as shown in Figure 6a. During the fluorescence titration, a significant increment in the fluorescence peak at 399 nm was noticed. The quenching of the emission central peak at 481 nm confirmed the chelation of mercury ions by the IXZD sensing probe. Further, the emission peak at 475 nm was significantly enhanced by gradually increasing [Hg(II)], and inset Figure 6a exhibits the dependence of the F_399_/F_481_ ratio versus [Hg(II)] that proved the complex formation. The reaction mechanism can be referred to as the internal charge transfer (ICT) mechanism [41]: IXZD acts as a donor molecule that chelates Hg(II) metal ions which act as a receptor. The IXZD sensor exhibited a high affinity to mercury with a 0 to 4 μM concentration range. The LOD was estimated from the fluorescence titration of IXZD versus Hg(II). Assuming that emission intensity can be identified with a precision of ±1%, the LOD is estimated to be 0.025 μM Hg(II) [42,43]. Therefore, the IXZD sensor significantly influences the chemical sensor for tracing mercury ions.

### 3.5. Sensitivity and Selectivity of IXZD

**Figure 7:**
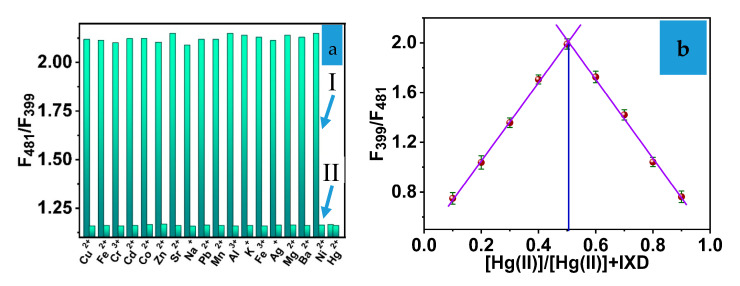
(**a**) IXZD emission spectra at 481 nm under 351 nm excitation (I) in the presence of interfering cations and (II) interfering cations and Hg(II). The IXZD: Hg(II) is 1:1; (**b**) Job’s plot for determination of the IXZD: Hg(II) stoichiometry.

The influence of different metal ions such as alkali, alkaline, and transition metal ions on the sensitivity of IXZD was studied. The interference experiments were carried out, the IXZD chemosensor fluorescence intensities were recorded, and the investigated interfering ions were added to the surrounding medium during the sensing procedure. The sensing efficacy of the optical sensor film to Hg(II) ions does not show any alterations due to the presence of other metal ions. Therefore, the attendance of the different cations to affect the binding model of Hg(II)-IXZD was examined in a 20 mM BR buffer medium using fluorescence intensity detections (Figure 7a). The interfering cations did not significantly interfere with the sensing mechanism of the chemosensor film-IXZD to mercury ions. Thus, the chemosensor film shows considerable sensitivity for tracing Hg(II).

The binding stoichiometric of the IXZD-Hg(II) chelation reaction was estimated using Job’s plot [44] based on the fluorescence intensities of IXZD as presented in Figure 7b. Job’s scheme was designed by varying the concentration of mercury ions (0:1 mole fraction) versus the fluorescence intensities ratio of IXZD sensing molecules. During the addition process of Hg(II), the emissions of the IXZD peaks at 399 and 481 nm were recorded. A 2.0 mL mixture with Hg(II) ions and IXZD chemical sensor with a molar ratio of (1:1) was applied in a buffered medium with a pH of 7.4 utilizing (20 mM BR solution). The resulting mixture has a 10 μM Hg(II)-IXZD. The emission ratio of 481/399 against the concentration Hg(II) exhibits the fluorescence IXZD emission taking place at 0.5 equivalent, providing that the stoichiometric of Hg(II): IXZD ratio is 1:1. The resulting data agree with the absorbance results.

### 3.6. Binding Reaction

The Benesi–Hildebrand equation was applied to investigate the binding reactivity of the IXZD sensing molecule to mercury ions. The binding constant of IXZD-Hg(II), depending on the fluorescence intensities data, was calculated:

$$\frac{1}{F-F_{o}}=\frac{1}{K_{b}\;\left(F_{max}-F_{o}\;\right)\;\left[Hg\left(II\right)\right]}+\frac{1}{F_{max\;}-F_{min}\;}.$$

**Figure 8:**
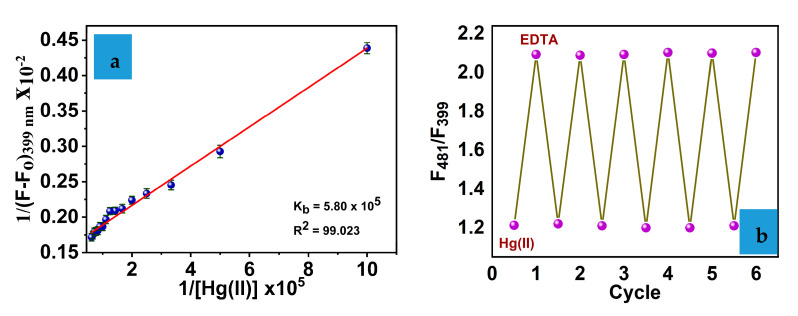
(**a**) Bensi–Hildebrand formulation based on (1/F–F_o_) versus Hg(II) (**b**) The reversibility of IXZD optical sensor using EDTA at λ_em_ = 481 nm.

*F_o_, F,* and *F_max_* are emission intensities of the IXZD at 399 nm detected in the absence of Hg(II) at various concentrations and a 1:1 equivalent molar ratio (IXZD: mercury ions), respectively, and where *K_b_* is the binding constant. The Benesi–Hildebrand equation estimates the *K_b_* to be 5.8 × 10^5^ M^−1^ (Figure 8a) [44].

Moreover, the recycling of the sensing affinity based on the binding of the IXZD-Hg(II) complex was investigated by the titration of the IXZD film versus ethylenediaminetetraacetic acid (EDTA) in the presence of Hg(II) [45,46,47]. Introducing EDTA to the surrounding medium with the sensor film after adding the Hg(II) ions increases the emission peak at 399 nm and subsequently decreases the 481 nm peak. This behavior can be attributed to the binding interaction of Hg(II) by EDTA, as shown in Figure 8b. This substitution reaction was confirmed by activating the free IXZD sensing molecule in the sensor film and enhancing the emission of 399 nm under 351 nm excitation. Thus, the IXZD optical film can be a characteristic sensor for tracing Hg(II) cations.

Table 1 summarizes the LOD values of the optical sensor-based organic molecules used in Hg(II) detection. The present work exhibits a great efficiency for detection of Hg(II) ions with respect to recent research-based organic molecules.

### 3.7. Theoretical Calculations

Density functional theory (DFT) and the time-dependent DFT (TDDFT) theoretical calculations were performed using the Gaussian09 program [57] to understand the electronic properties of the ligand alone, the ligand in the presence of the H_3_O^+^ molecule, and the ligand forming a complex with Hg^2+^. These electronic properties contribute to ESIPT properties [58] and the quenching of ESIPT fluorescence. The PBE1PBE functional and 6-31G(d)/Lanl2dz basis sets were applied to optimize the different structures. Hg atoms were treated by LANL2DZ basis set, and all other atoms were treated by 6-31G(d). The solvation model SMD and water were included in all the calculations. We performed vibrational frequency calculations at the same theory level to confirm the complex stability with the IXZD chemical probe.

**Figure 9:**
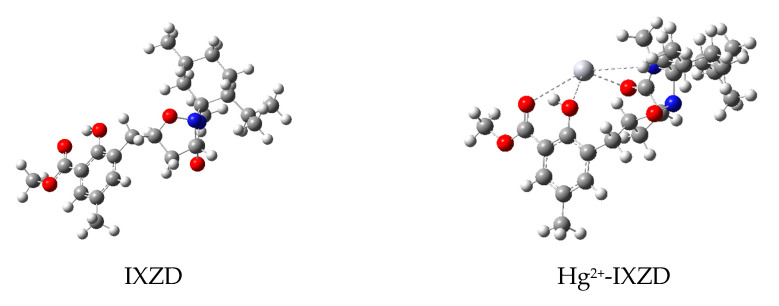

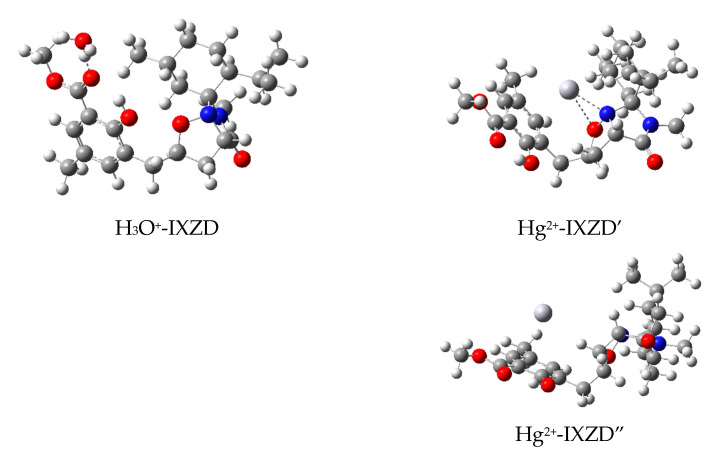
Complexes L, H_3_O^+^-L, and Hg^2+^-L models optimized by DFT with solvation model SMD and water.

Using the optimized structures, we have applied TDDFT calculations with solvation model SMD and water through the PBE1PBE/ aug-cc-pVDZPP/6-31+G(d) level of theory. Dunning’s double zeta augmented correlation consistent polarized basis set (aug-cc-pVDZPP) was used for Hg along with the corresponding effective core potentials (ECP) [59,60]. The optimized structures with solvation model SMD and water of the ligand alone (IXZD), the ligand in the presence of the H_3_O^+^ molecule (H_3_O^+^- IXZD), and the Hg^2+^ complexes (Hg^2+^- IXZD) are represented in Figure 9. The relative energies for the different Hg^2+^ complexes are given in Table 2.

We show in Figure 9 that Hg^2+^ can have short interactions with three oxygens and one nitrogen of the ligand, as in Hg^2+^-IXZD. We propose that this structure is the reference as it has the lowest energy among the other conformations. In Hg^2+^-IXZD’, the Hg has two short interactions with one nitrogen and one oxygen belonging to the ring of the ligand and two long interactions with two oxygens. The relative energy for this compound is 1.8 kcal mol^−1.^ In Hg^2+^-IXZD”, we have only two long interactions with two oxygens of the ligand. The relative energy for this last compound is 8.2 kcal mol^−1^.

**Figure 10:**
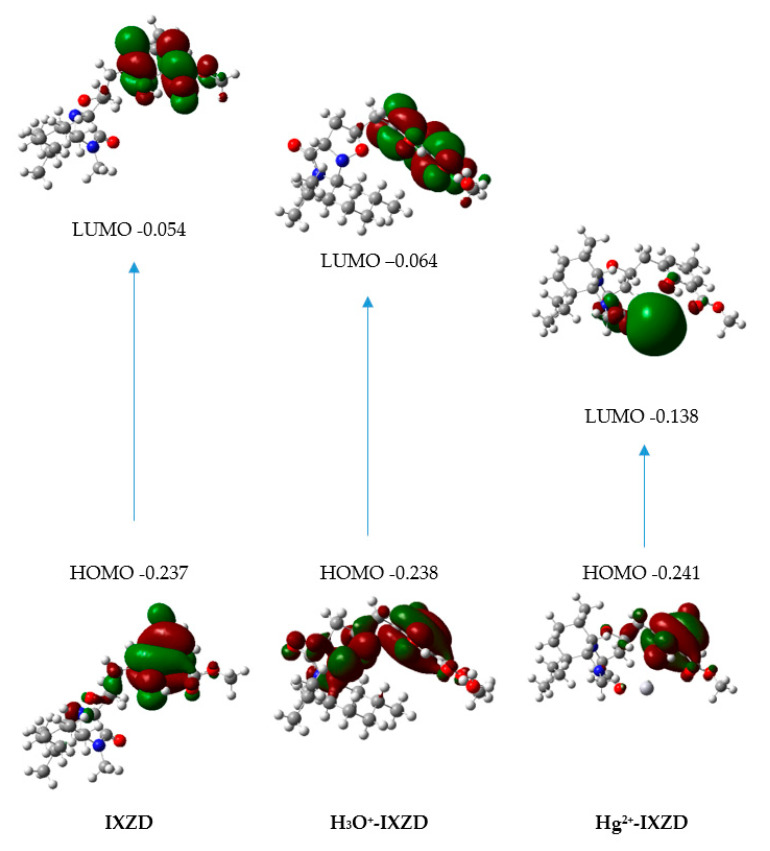
Frontier orbitals and their energies for L, H_3_O^+^-IXZD, and Hg^2+^-IXZD.

The possible transitions were calculated for the most stable structures by performing time-dependent DFT. The selected molecular orbitals of IXZD, H_3_O^+^-IXZD, and Hg^2+^-IXZD are presented in Figure 10. TDDFT data involving electronic transitions, wavelength, and oscillator strength are found in Table 3. The electron density in the L molecule is mainly detected on the aromatic ring. For the charge redistribution about the hydrogen-bonded parts, the hydroxyl moiety contributes largely to HOMO, while its contribution decreases in LUMO. It indicates that the electron density shifts from the hydroxyl group to the adjacent oxygen upon photoexcitation of IXZD from state S_0_ to state S_1_, which provides the tendency for ESIPT reaction [46].

In H_3_O^+^-IXZD, we see a similar effect in the electronic transition in IXZD. The HOMO is now more delocalized on the whole molecule. This results in a lower value of the oscillator strength for this transition (0.133 for H_3_O^+^-IXZD compared to 0.158 for IXZD), showing less electron density transfer from the hydroxyl group to the adjacent oxygen. This results in less tendency for ESIPT reaction and contributes to the quenching of the ESIPT-based fluorescence probe. This suggests that this effect is enhanced in the presence of more H_3_O^+^ molecules when the medium is more acidic. In Hg^2+^-IXZD, the transition from HOMO to LUMO offers an intramolecular charge transfer (ICT): the electron density in the HOMO is mainly on the hydroxyl group and the aromatic ring of the ligand IXZD, but the electron density in the LUMO is essentially distributed on the metal Hg. There is no shift of the electron density from the hydroxyl group to the adjacent oxygen, which blocks the tendency for ESIPT reaction. Thus, the ICT from the IXZD molecule to Hg(II) contributes to the quenching of the ESIPT-based fluorescence probe. These results are supported by the low value of the oscillator strength (Table 3).

## 4. Conclusions

The IXZD sensing molecule was designed and developed as an enormously selective chemosensor for tracing Hg(II). The properties of the IXZD molecule are based on UV–Vis absorbance spectra, and fluorescence spectra were examined in the presence of mercury ions using a 20 mM BR buffer system at an adjusted pH medium. The IXZD molecule was embedded in a PVC film. The fabricated sensing film exhibits excellent selectivity and sensitivity to mercury ions. The detection limit was determined to be 0.025 mM Hg(II) concentration without showing any interference with the presence of any cations in the surrounding medium. Additionally, Hg(II) ions affect the IXZD solution rapidly, decreasing the fluorescence peak at 481 nm and enhancing the fluorescence of a new fluorescence peak at 399 nm. Herein, we utilized Job’s plot to investigate that IXZD binds to Hg(II) ions with a ratio of 1:1. The proposed mechanism of sensing molecule is based on the binding of Hg(II) by IXZD molecule in the solution vicinity and producing an IXZD-Hg(II) complex. Density functional theory (DFT) and the time-dependent DFT (TDDFT) theoretical calculations were provided to study the Hg(II) complexation structures and their electronic properties in solution. The formation constant of the formed complex was calculated using the Benesi–Hildebrand equation. Lastly, the synthesized IXZD chemosensor could be used efficiently to detect Hg(II) ions.

## Figures and Tables

**Table 1:** Comparison of the IXZD sensor with recently research-based Hg(II) detection.

Sensor	LOD (M)	References
Rhodamine B	1.34 × 10^−6^	[48]
Rhodamine B + Polymer	0.05 × 10^−6^	[49]
BODIPY	0.77 × 10^−6^	[50]
Thiourea	0.1 × 10^−6^	[51]
Crown-ether	0.1 × 10^−6^	[52]
Coumarine	0.29 × 10^−6^	[53]
Pyrene	1.74 × 10^−6^	[54]
Anthracene	3.2 × 10^−6^	[55]
Oxadiazole	1.5 × 10^−6^	[56]
IXZD molecule	0.025 × 10^−6^	Present work

**Table 2:** Values of relative energies of the Hg^2+^ Complexes with solvation model SMD and water.

Complex	Relative Energy (kcal/mol)
Hg^2+^-IXZD	0
Hg^2+^-IXZD’	1.8
Hg^2+^-IXZD”	8.2

**Table 3:** TDDFT data of selected electronic transitions, absorption energies, and oscillator strengths.

Complex	Transition	λ(nm)	Oscillator Strength
IXZD	HOMO -> LUMO	303	0.1584
H_3_O^+^-IXZD	HOMO -> LUMO	318	0.1338
Hg^2+^-IXZD	HOMO -> LUMO	599	0.0404

## Data Availability

Not applicable.

## Supplementary Materials

The following supporting information can be downloaded at: https://www.mdpi.com/article/10.3390/bios12111028/s1, Figure S1: ^1^H spectrum of the synthesized compound. Figure S2: ^13^C spectrum of the synthesized compound.
