# Supplementary material for: A Reversible Optical Sensor Film for Mercury Ions Discrimination Based on Isoxazolidine Derivative and Exhibiting pH Sensing
Source: Biosensors (Basel). 2022 Nov 16;12(11):1028. doi: 10.3390/bios12111028 (PMC9688351; doi:10.3390/bios12111028)
Supplement: Supplementary file 1 [file biosensors-12-01028-s001.zip › biosensors-2028749-supplementary.pdf]

A novel reversible chemosensor for discrimination of Hg(II) in aqueous solution based on isoxazolidine derivative and exhibiting pH sensing attendance.

NMR of the synthesized compound:

NMR ^{1}H (800 MHz, $CDCl_3$) δ_{ppm}: 0.65 (d, 3H, *J*= 6.4 Hz, CH_3), 0.80 (d, 3H, *J*= 6.4 Hz, CH_3), 0.82 (d, 3H, *J*= 6.4 Hz, CH_3), 0.83 (m, 1H), 1.09 (t, 1H, *J* = 11.2 Hz), 1.21 (m, 1H), 1.28 (m, 1H), 1.36 (quin, 1H, J = 6.4 Hz), 1.56 (m, 1H), 1.70 (m, 1H), 1.74 (m, 1H), 1.87 (m, 1H), 2.19 (s, 3H, CH_3), 2.21 (ddd, 1H, J = 2.4, 8.0 and 12 Hz, H4a), 2.61(dd, 1H, J = 4.8 and 12 Hz, H6), 2.65 (s, 3H, NCH_3), 2.69 (ddd, 1H, J = 5.6, 8.0 and 12 Hz, H4b), 3.01 (dd, 1H, J = 4.8 and 12 Hz, H6), 3.88 (s, 3H, OCH_3), 3.96 (m, 2H, H3 and H5), 7.17 (d, 1H, J = 1.6 Hz, H_{Ar}), 7.47 (d, 1H, J = 1.6 Hz, H_{Ar}), 10.75 (s, 1H, OH). NMR ^{13}C (200 MHz, $CDCl_3$) δ_{ppm}: 18.3, 20.2, 21.8, 22.2, 24.0, 24.2, 25.9, 29.4, 32.5, 34.4, 38.5, 40.3, 47.9, 52.1, 66.2, 76.4, 89.7, 111.4, 126.1, 127.5, 127.9, 138.1, 157.4, 170.8, 172.6. Anal. Calcd. for $C_{25}H_{36}N_2O_5$ (444.57): C, 67.54; H, 8.16; N, 6.30, Found: C, 67.77; H, 8.22; N, 6.39.

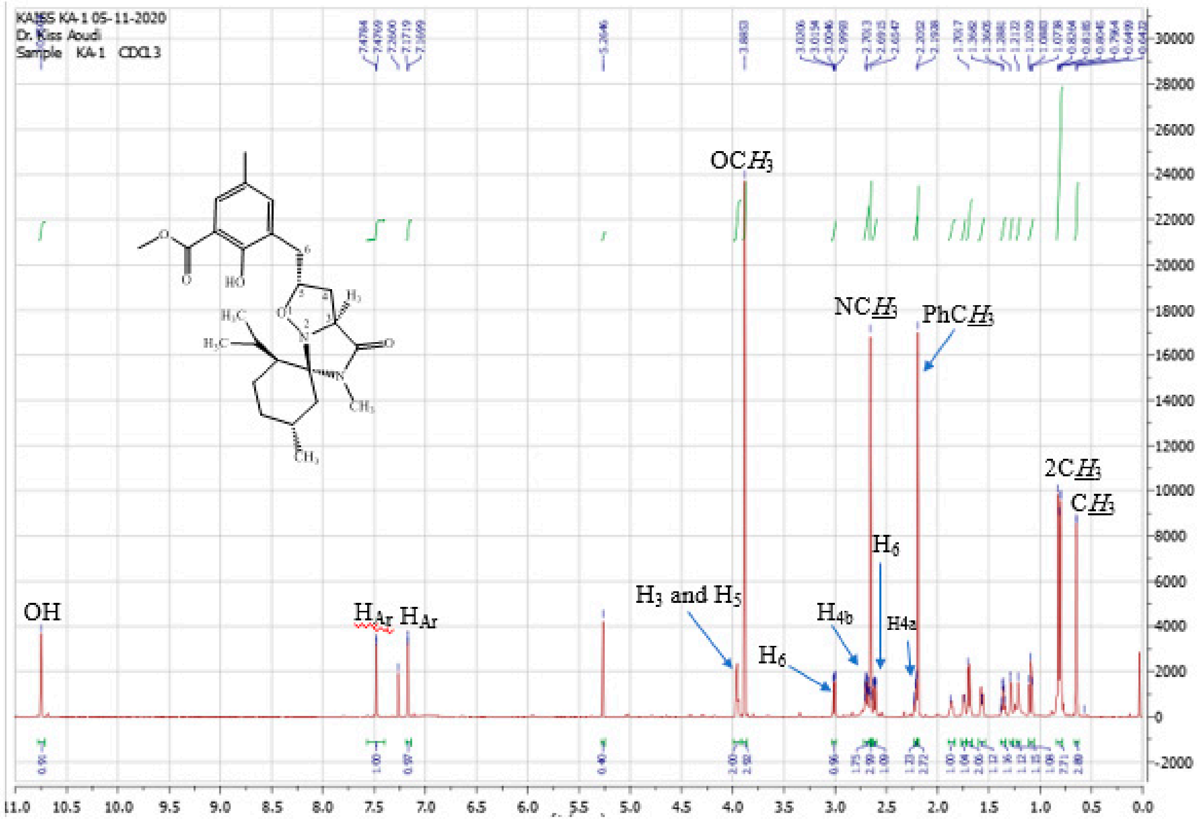

Figure S1. ^{1}H spectrum of the synthesized compound.

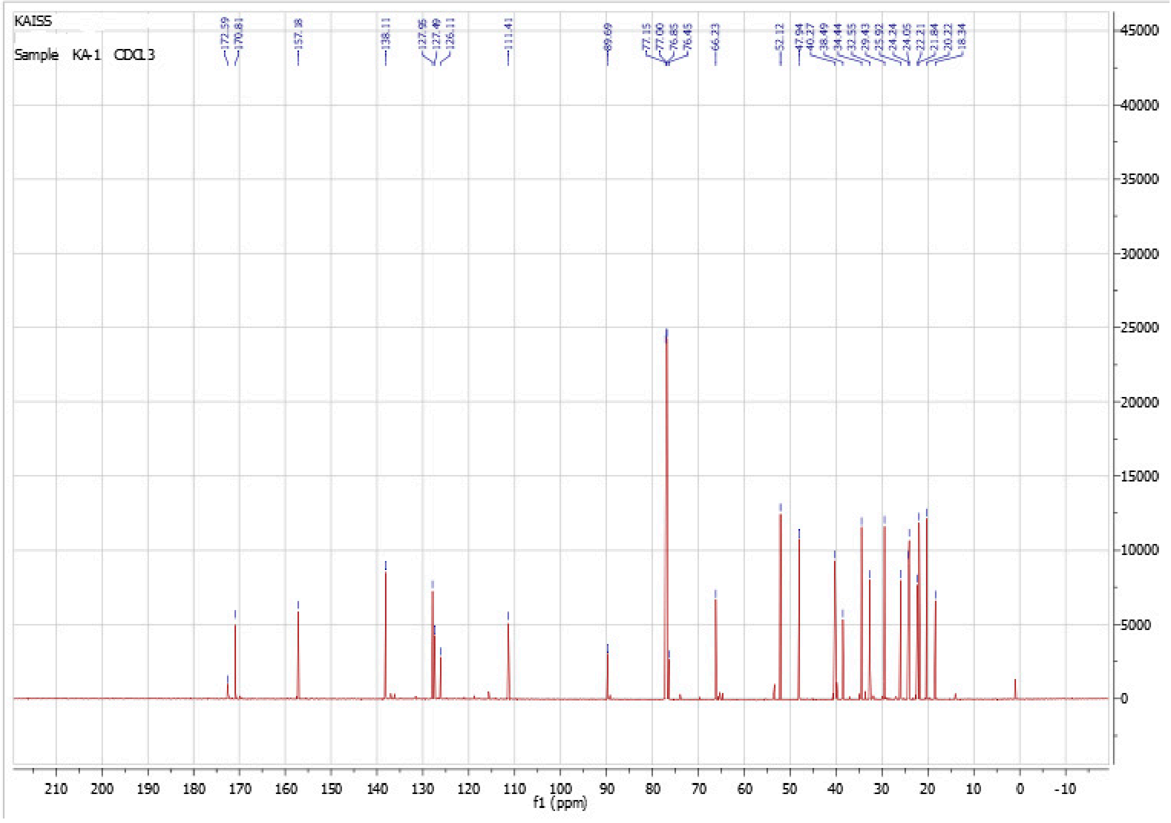

Figure S2. ^{13}C spectrum of the synthesized compound.
